# Supplementary material for: The genome sequence of the European Pepper moth, Duponchelia fovealis (Zeller, 1847) (Lepidoptera: Crambidae)
Source: Wellcome Open Res. 2026 Feb 9;11:96. [Version 1] doi: 10.12688/wellcomeopenres.25824.1 (PMC13040225; doi:10.12688/wellcomeopenres.25824.1)
Supplement: Peer review history

# Reviewer report: Reviewer response for version 1

**DOI:** 10.21956/wellcomeopenres.28425.r150039

**Published:** 2026-03-31

**Authors:** Gábor Sramkó

**Affiliations:** 1 University of Debrecen, Debrecen, Hungary

This genome report reports the high-quality (chromosome-level) assembly of a crambid moth, which is a significant pest in some areas.

 As a genome report, it is descriptive and serves the purpose of characterizing the assembled genome and how it was obtained through wet-lab data generation and subsequent bioinformatic steps. As such, it is a complete and well-written report, and I recommend accepting it after two minor corrections of typos, both in the paragraph commencing with "Most of the assembly sequence (99.95%) was assigned...":

 1.) The Z & W chromosomes wer identified... -> The Z & W chromosomes were identified...

 2.) (13.8 - 21.9 Mbp) are unknown.. <- here, please, remove the second full-stop.

Are sufficient details of methods and materials provided to allow replication by others?

Yes

Is the rationale for creating the dataset(s) clearly described?

Yes

Are the datasets clearly presented in a useable and accessible format?

Yes

Are the protocols appropriate and is the work technically sound?

Yes

Reviewer Expertise:

Phylogenomics, population genomics, and conservation genomics that include genome assembly.

I confirm that I have read this submission and believe that I have an appropriate level of expertise to confirm that it is of an acceptable scientific standard.

# Reviewer report: Reviewer response for version 1

**DOI:** 10.21956/wellcomeopenres.28425.r150704

**Published:** 2026-03-25

**Authors:** Hans-Peter Fuehrer

**Affiliations:** 1 University of Veterinary Medicine, Veinna, Austria

The authors present the genome sequence of the European pepper moth. The assembly contains 2 haplotypes with total lengths of 466.58 MBs and 414.21 MBs The manuscript is of good quality.

 It is recommended to re-word the term “crambid pest”

 Moreover, to clarify, Southern Europe includes (most parts of) the Mediterranean area

Are sufficient details of methods and materials provided to allow replication by others?

Yes

Is the rationale for creating the dataset(s) clearly described?

Yes

Are the datasets clearly presented in a useable and accessible format?

Yes

Are the protocols appropriate and is the work technically sound?

Yes

Reviewer Expertise:

Medical and veterinary entomology

I confirm that I have read this submission and believe that I have an appropriate level of expertise to confirm that it is of an acceptable scientific standard.
